# Noninvasive PPROM risk stratification with explainable AI using routine antenatal CRP and albumin

**DOI:** 10.1038/s41746-026-02884-z

**Published:** 2026-06-10

**Authors:** Shidong Tan, Sihan Wu, Shengnan Wu, Yicheng Zhou, Yamin Li, Hang Su, Shujian Zhang, Yao Xu, Xiaoying He, Yanqiang Han, Liping Jin, Jinjin Li, Zhiyun Wei

**Affiliations:** 1 Clinical and Translational Research Center, Department of Integrated Traditional Chinese Medicine (TCM) and Western Medicine, Shanghai Key Laboratory of Maternal Fetal Medicine, Shanghai Institute of Maternal Fetal Medicine and Gynecologic Oncology, Shanghai First Maternity and Infant Hospital, School of Medicine, Tongji University, Shanghai, China; 2 Obstetrics & Gynecology Hospital of Fudan University, Center of Big Data & Biobank, Shanghai Key Lab of Reproduction and Development, Shanghai Key Lab of Female Reproductive Endocrine Related Diseases, Shanghai, China; 3 School of Computer Science, Hubei University, Wuhan, China; 4 National Key Laboratory of Advanced Micro and Nano Manufacture Technology, Shanghai Jiao Tong University, Shanghai, China; 5 School of Integrated Circuits (School of Information Science and Electronic Engineering), Shanghai Jiao Tong University, Shanghai, China; 6 Inner Mongolia Research Institute, Shanghai Jiao Tong University, Hohhot, China; 7 Shanghai Jincheng Technology Co. Ltd, Shanghai, China; 8 State Key Laboratory of Biocatalysis and Enzyme Engineering, Hubei Key Laboratory of Industrial Biotechnology, School of Life Sciences, Hubei University, Wuhan, China

**Keywords:** Biomarkers, Computational biology and bioinformatics, Diseases, Health care, Medical research, Risk factors

## Abstract

Premature rupture of membranes (PROM) and preterm PROM (PPROM) are significant obstetric complications, affecting 10–20% and 3% of pregnancies globally and constituting a major cause of preterm birth and neonatal morbidity. While intrauterine infection is a key underlying driver, timely identification of high-risk cases remains a clinical challenge. We developed a noninvasive artificial intelligence (AI) predictive framework using a large-scale dataset of 114,601 antenatal laboratory and ultrasound measurements. Our multimodal model integrated longitudinal clinical measures, including maternal demographics, laboratory results, and ultrasound data. The optimized XGBoost algorithm achieved high performance (AUC: 0.952) and identified elevated C-reactive protein (CRP) and decreased albumin levels in late pregnancy as top features, underscoring the critical roles of inflammation and oxidative stress. SHAP analysis ensured interpretability, unveiling dynamic risk patterns across gestation. This AI-driven approach provides a precise, noninvasive tool for late-gestation PPROM risk stratification, paving the way for translation into precision obstetric care.

## Introduction

Premature Rupture of Membranes (PROM) is a common obstetric complication characterized by spontaneous membrane rupture before labor onset, with a global incidence ranging from 10% to 20%^1^. It may occur at term (≥37 weeks) or earlier (<37 weeks, termed preterm Premature rupture of membranes, PPROM). PPROM complicates approximately 3% of all pregnancies and contributes to 30–40% of preterm births, significantly impacting neonatal morbidity and mortality^2,3^.

The systemic risks associated with PPROM are multifaceted. Membrane rupture exposes the intrauterine environment to ascending pathogens, elevating infection risks^4^. Clinical infections manifest in 12–15% of PPROM cases postpartum, with chorioamnionitis (CAM) being a hallmark complication characterized by maternal fever, tachycardia, and uterine tenderness; untreated CAM may progress to sepsis^5^. Notably, CAM incidence rises significantly from 2.7% to 11.8% when membrane rupture exceeds 12 h^4^. Prolonged PPROM before fetal viability (<24 weeks) elevates risks of specific fetal morbidities such as pulmonary hypoplasia and fetal distress^6^. Subclinical infections can trigger fetal tachycardia, intrauterine hypoxia, or stillbirth, while mechanical complications like cord prolapse or malpresentation further exacerbate fetal hypoxia, often necessitating emergent cesarean delivery^1,5^. Maternal risks encompass conditions such as placental abruption and catastrophic events like amniotic fluid embolism (AFE)^7^. It should be noted that while the most common causes of maternal mortality globally are hemorrhage, hypertensive disorders, and infection/sepsis, AFE—due to its rapid onset and poor prognosis—is a rare but highly lethal obstetric emergency^8^. Intrauterine infection is recognized as a critical driver of adverse outcomes in PPROM. The causal relationship between infection and PPROM, coupled with the nonspecific nature of early symptoms, complicates timely diagnosis. Conventional biomarkers such as leukocyte count and C-reactive protein (CRP) lack sensitivity for early infection detection, often delaying treatment initiation. Once established, intrauterine infections trigger rapid bacterial proliferation, toxin release, and cytokine storms, exacerbating uterine dysfunction and worsening prognosis. Therefore, the timely identification of high-risk pregnancies and proactive management prior to complications are critically important.

However, current predictive models for PPROM remain limited by late detection and invasiveness. While novel biomarkers like matrix metalloproteinase-8 and -9 (MMP-8/9) show diagnostic promise^2,9^, their clinical utility is hindered by small validation cohorts, standardization challenges, cost constraints, and suboptimal warning windows. This underscores an urgent need for noninvasive, cost-effective, and highly specific methods to enable improved risk assessment in PPROM.

Artificial Intelligence (AI), encompassing systems designed to mimic human intelligence through perception, reasoning, learning, planning, and prediction, offers transformative potential in healthcare. Machine learning (ML) and deep learning (DL) algorithms excel at resolving high-dimensional collinearity and feature redundancy by extracting complex nonlinear patterns from large-scale clinical datasets. These techniques have demonstrated superior performance over conventional methods in medical imaging analysis, disease risk stratification^10^, and personalized treatment optimization^11^. For instance, the TIMES scoring system integrates spatial immune profiles to predict hepatocellular carcinoma recurrence with 82.2% accuracy^12^, the CRFC model employs random forest algorithms for high-precision respiratory virus infection forecasting using environmental data^13^, and ML models applied to electronic health records (EHR) enable dynamic risk assessment for conditions like acute upper gastrointestinal bleeding shortly after admission, potentially reducing unnecessary hospitalizations^14^. These advancements highlight AI’s potential to enhance predictive accuracy and therapeutic decision-making in precision medicine.

Applying AI to PPROM risk stratification addresses key limitations of traditional approaches. Conventional biomarker analysis often suffers from biological variability in single indicators and subjectivity in threshold determination. AI can overcome these constraints by integrating multimodal data—including demographic characteristics, laboratory results, and ultrasound parameters—to detect subtle pathophysiological changes associated with increased risk. Moreover, PPROM pathogenesis involves dynamic interactions among inflammatory cytokine cascades, matrix metalloproteinase activity regulation, and biomechanical membrane alterations; AI’s capacity to model high-dimensional nonlinear relationships aligns precisely with this complexity. AI models also possess iterative learning capabilities, allowing continuous refinement through new data integration—a foundation for developing population-specific dynamic risk stratification systems.

A recognized challenge with increasingly complex AI models is the “black box” dilemma, where interpretability is compromised. To bridge this gap and foster clinical trust, Explainable AI (XAI) techniques are increasingly adopted in medicine. This study incorporates SHAP (SHapley Additive exPlanations), a game theory-based interpretability framework, to quantify feature contributions to classification. SHAP provides both global feature importance rankings and sample-specific explanations, delineating how individual variables influence risk stratification. Through SHAP-derived visualizations like beeswarm plots, clinicians gain intuitive insights into model decision-making processes, thereby enhancing trust in AI-driven clinical guidance.

Leveraging 114,601 clinical samples from Shanghai First Maternity and Infant Hospital, this study establishes a risk assessment model using routine antenatal indicators across all gestational stages. By focusing on the clinically critical window preceding typical PPROM onset, the model achieves an accuracy of 0.922. Integrated with SHAP interpretability, it forms a closed-loop “risk assessment-interpretation” system. This approach demonstrates cost-effectiveness and improved patient compliance, offering a novel paradigm for late-gestation imminent PPROM risk stratification and precision care.

## Results

### Data source and processing

**Fig. 1:**
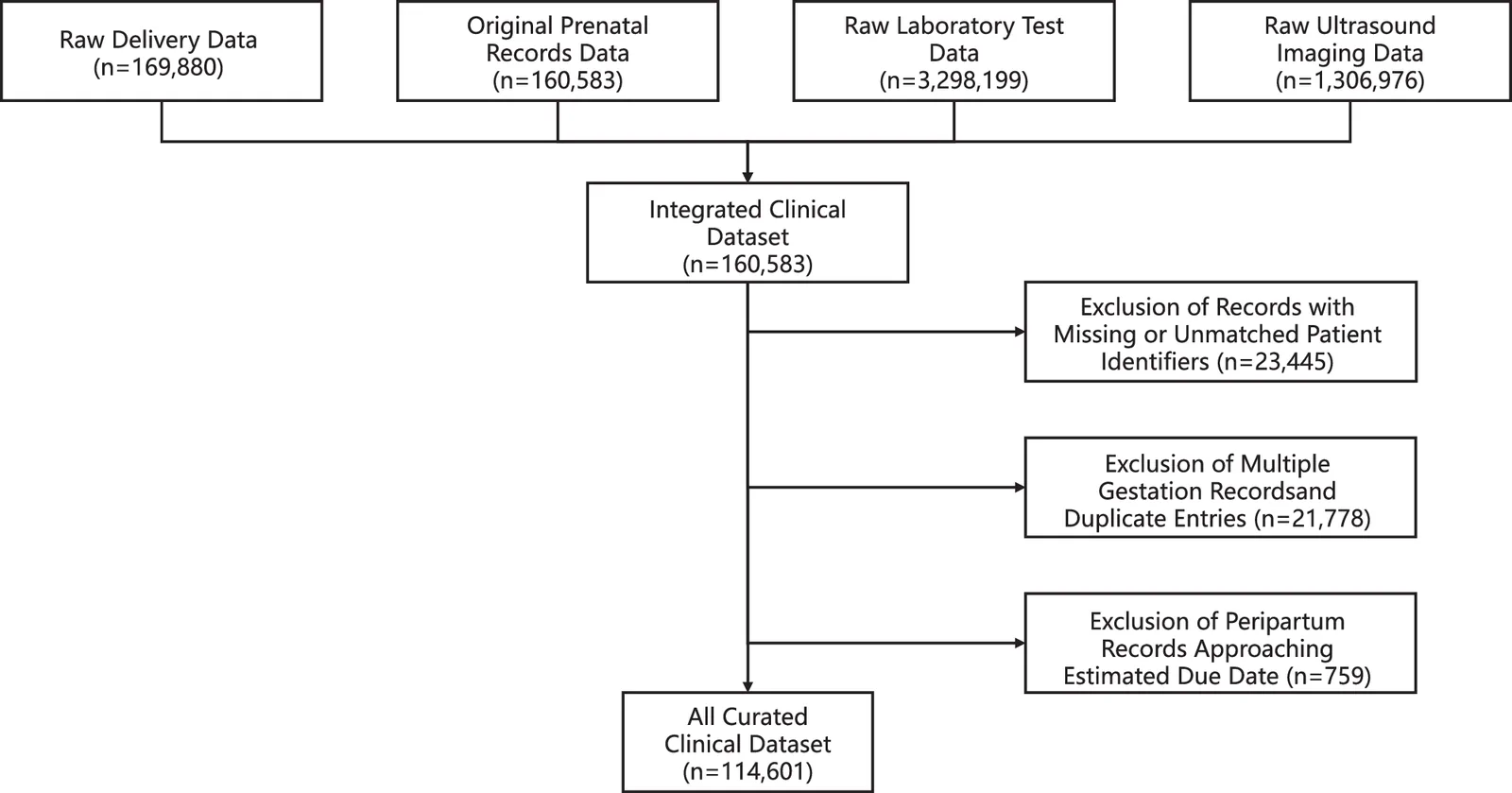
Flowchart of clinical dataset acquisition. Multisource medical records were integrated using identifiers, followed by rigorous exclusion of records not meeting inclusion criteria, establishing the final curated cohort.

**Fig. 2:**
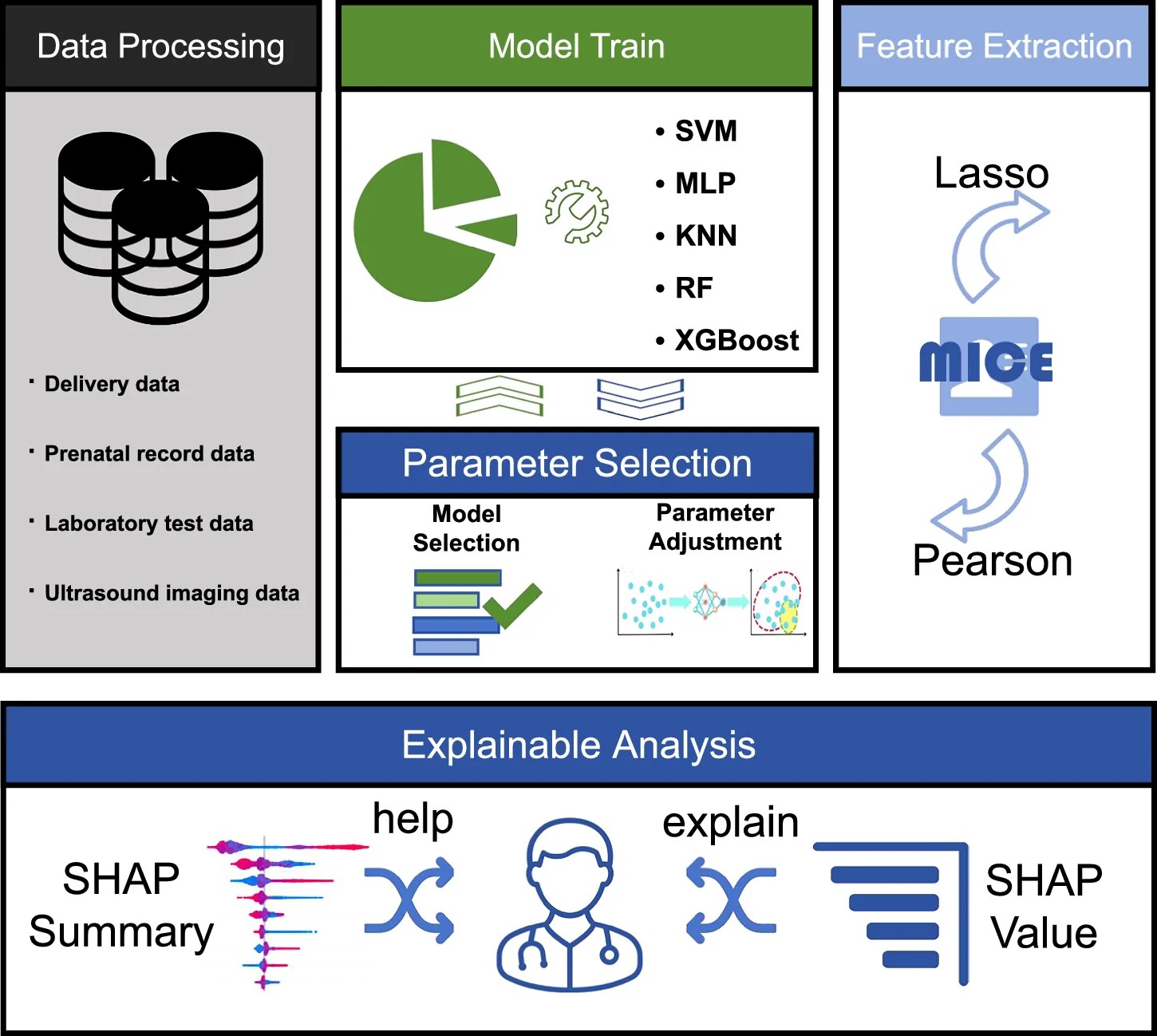
Data processing, modeling, interpretability analysis flowchart. In the process of establishing a risk stratification model, the data is processed, then the model is trained and selected. The superior model is selected according to the indicators and further optimized. The feature importance is obtained in the iterative process.

This study utilized data from a cohort of singleton pregnancies that initiated prenatal care at Shanghai First Maternity and Infant Hospital (2013–2019). Through identifier-linked integration of multisource medical records—including initial registration (maternal demographics: age, nationality, occupation), delivery documentation (intrapartum/postpartum parameters, neonatal weight), prenatal laboratory tests (serological analyses), and ultrasonography measurements (fetal head circumference)—we consolidated patient data. After rigorous exclusion of cases not meeting inclusion criteria (multiple gestations [*n* = 21,778]; irreconcilable key data [*n* = 23,445]; indeterminate perinatal outcomes [*n* = 759]), a final cohort of 114,601 eligible pregnancies was established (Fig. 1). Subjects were stratified by clinical diagnosis of term premature rupture of membranes (PPROM) into a PPROM cohort (*n* = 1895) and a term non-PPROM control cohort (*n* = 112,706) for comparative analysis (Fig. 2).

### Model performance

**Fig. 3:**
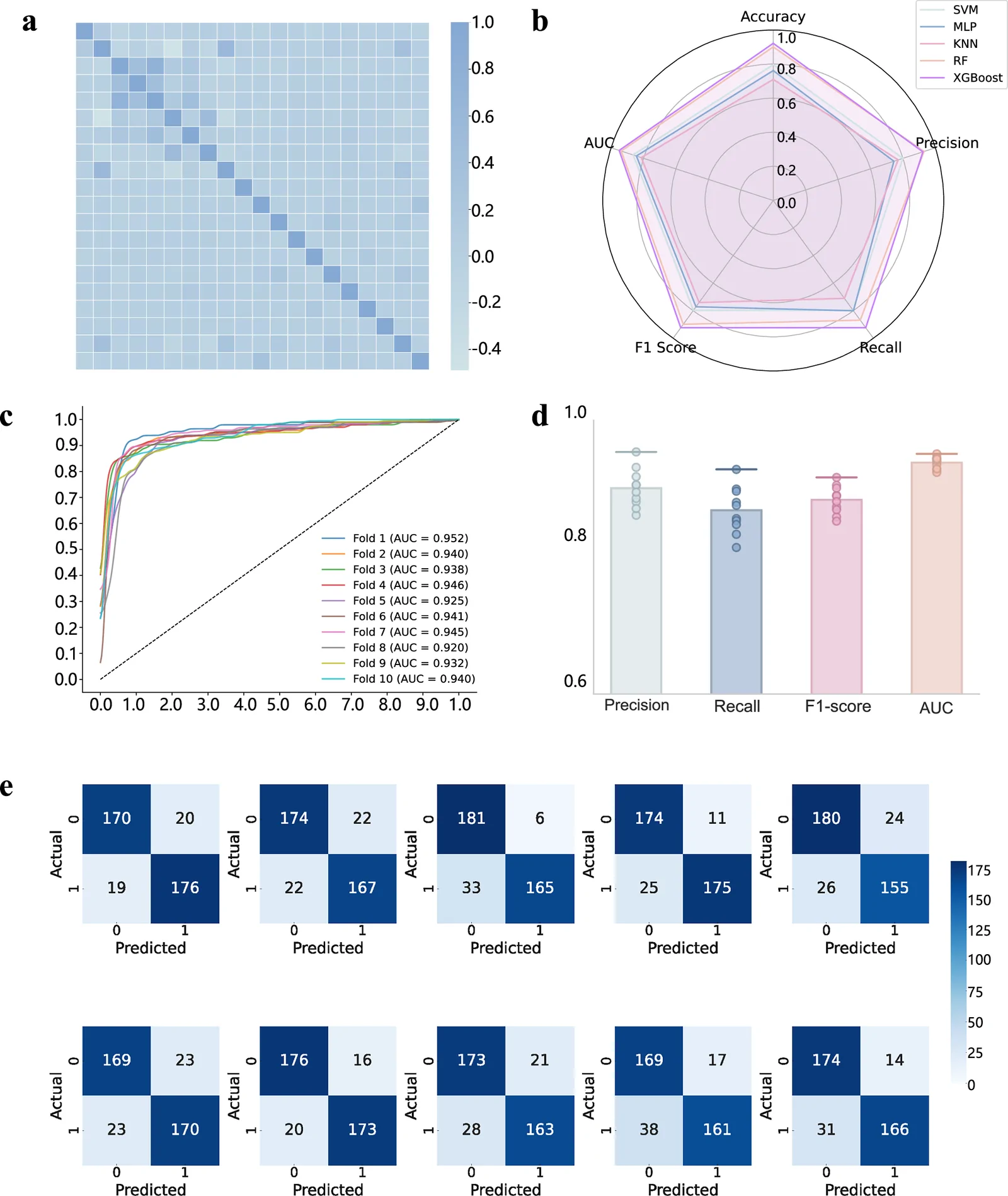
Comprehensive evaluation of model performance and feature correlation. **a** Pearson’s correlation coefficient matrix between features of top 20 importance. **b** Radar charts of indicators for the five base models in test sets. **c** ROC curves on 10-fold test datasets of XGBoost model. **d** Box plots of each metric on XGBoost model for 10-fold test datasets. **e** Confusion matrixes on 10-fold test datasets.

Model selection and optimization: Comparative analysis of five candidate models across ten test datasets (evaluated by AUC, accuracy, precision, recall, and F1-score), where predicted probability higher than 0.50 is defined as positive, demonstrated XGBoost’s superior performance in PPROM classification (Fig. 3 and Table 1). We therefore selected XGBoost as our final risk stratification model. Hyperparameter Tuning: Using Bayesian optimization (200 iterations) with five-fold cross-validation, we identified the hyperparameter combination maximizing AUC. The optimized model achieved: AUC: 0.952, Accuracy: 0.922, Precision: 0.923, Recall: 0.923, F1-score: 0.923 in test sets (Fig. 3c). Validation Performance: The mean AUC across all ten test datasets was 0.938 (Fig. 3d), confirming model robustness. Test-set confusion matrices further demonstrated generalizability (Fig. 3e). The calibration curve demonstrated close alignment with the ideal line (Brier score = 0.051), indicating well-calibrated probability estimation (Fig. S1). These results indicate high discriminatory power for PPROM risk stratification.

**Table 1:** Comparison of parameters between PPROM and Non-PPROM groups (Top 20 characteristics shown)

Feature	PPROM	Non-PPROM	*P* value
C-reactive protein (34–36^+6^ w)(mg/L)	5.8345 ± 2.8280	4.0424 ± 1.9802	<0.001
Albumin (34–36^+6^ w)(g/L)	34.7389 ± 2.9384	36.8223 ± 2.2830	<0.001
Blood white blood cells (34–36^+6^ w)(10^9^/L)	10.3224 ± 3.2374	8.5191 ± 1.9143	<0.001
Total protein (34–36^+6^ w)(g/L)	61.2296 ± 4.8973	64.0418 ± 3.8594	0.031
Prealbumin (34–36^+6^ w)(mg/L)	195.3998 ± 36.0229	208.8135 ± 31.8063	<0.001
Lymphocyte percentage (34–36^+6^ w)(%)	17.2184 ± 5.7842	19.4237 ± 4.6853	<0.001
Absolute value of monocytes (34–36^+6^ w)(10^9^/L)	0.5863 ± 0.2129	0.4963 ± 0.1637	<0.001
C-reactive protein (28–33^+6^ w)(mg/L)	4.3751 ± 2.1957	4.0286 ± 1.9577	<0.001
Umbilical artery S/D (18^+1^–25 w)	3.0095 ± 0.4837	3.0424 ± 0.4501	0.002
Total protein (25^+1^–33^+6^ w)(g/L)	64.0819 ± 3.8641	64.2595 ± 3.5773	0.031
Urine specific gravity (35^+1^–36^+3^ w)	1.0159 ± 0.0055	1.0158 ± 0.0067	0.4201
Albumin (25^+1^–33^+6^ w)(g/L)	36.8925 ± 2.4628	37.2130 ± 2.1354	<0.001
Uric acid (25^+1^–33^+6^ w)(umol/L)	244.5204 ± 53.2335	236.1413 ± 48.6059	<0.001
Umbilical artery S/D (25^+1^–32 w)	2.4944 ± 0.4069	2.5479 ± 0.3750	<0.001
Blood white blood cells (20^+1^–27^+6^ w)(10^9^/L)	9.9404 ± 2.0936	9.6181 ± 1.9840	<0.001
Lymphocyte percentage (28–33^+6^ w)(%)	18.0880 ± 4.5128	18.1648 ± 4.1413	0.421
Urine pH (35^+1^–36^+3^ w)	6.6892 ± 0.5274	6.6731 ± 0.5275	0.1842
1 h glucose (mmol/L)	7.8399 ± 1.6088	7.5353 ± 1.5364	<0.001
Total cholesterol (25^+1^–33^+6^ w)(mmol/L)	6.1593 ± 0.7213	6.1493 ± 0.6448	0.4990
Alkaline phosphatase (25^+1^–33^+6^ w)(U/L)	225.7768 ± 49.6624	221.4256 ± 45.7149	<0.001

### Interpretability analysis

**Fig. 4:**
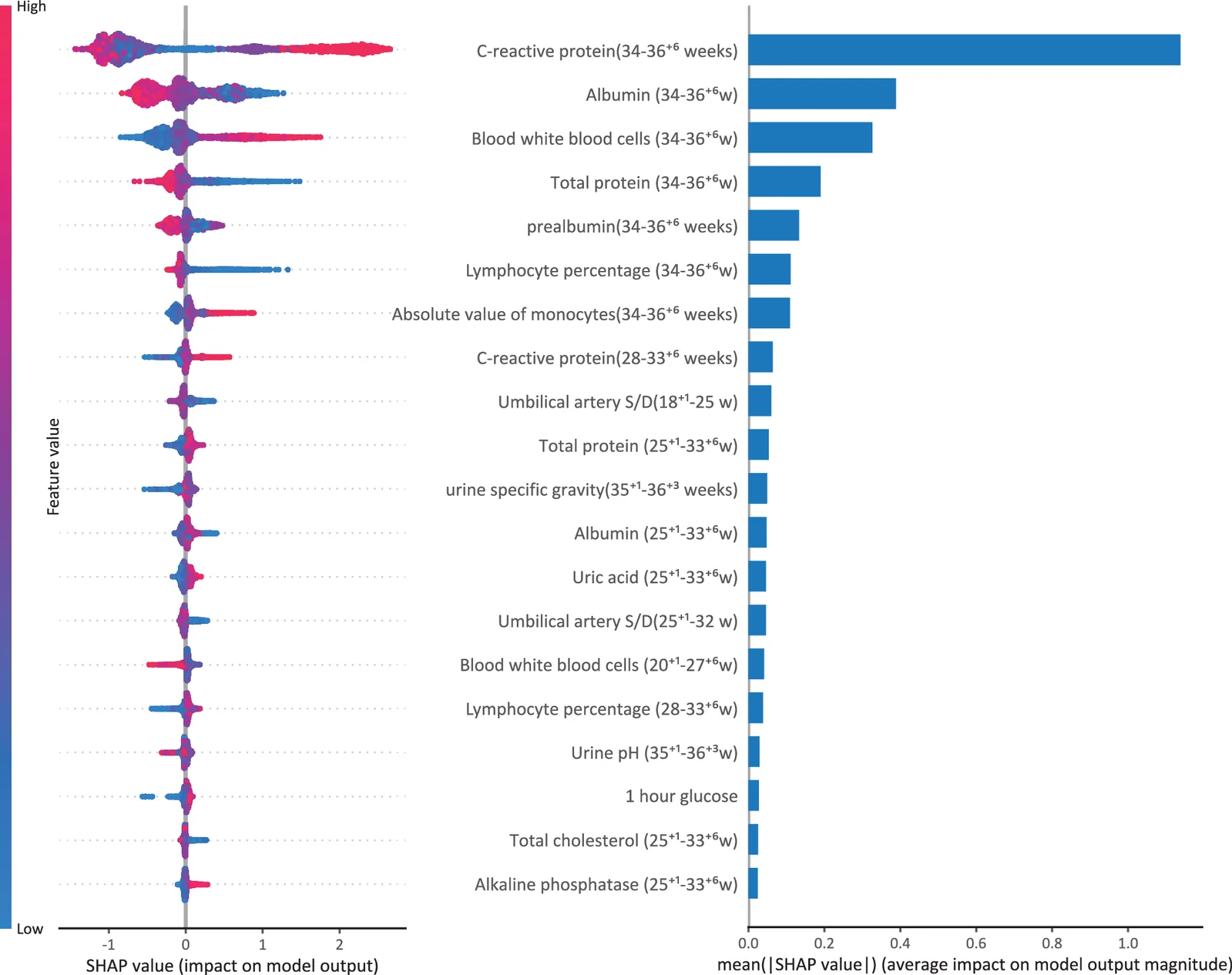
SHAP analysis for model explanation. The left and right panels show the SHAP swarm plot and feature importance of top 20 features, respectively.

We embedded SHAP interpretable analysis into the model training pipeline. During training, we ranked the global importance of the 75 input features using SHAP and highlighted the top 20 features. In addition, we plotted the SHAP beeswarm plot (Fig. 4), in which variables positioned higher indicate a stronger influence on model output. Each red or blue dot represents the contribution of a given feature for an individual sample: a SHAP value > 0 means the feature favors PPROM classification for that case, whereas a negative value indicates assignment to the non-PPROM group. This visualization illustrates the overall importance distribution of each feature for PPROM risk stratification and its directional effect on model output. The results of the SHAP importance ranking show that 34–36 ^+ 6^ weeks C-reactive protein and 34–36 ^+ 6^ weeks albumin play a crucial role in influencing the occurrence of PPROM.

The SHAP summary plot shows that higher CRP levels at 34–36 ^+ 6^ weeks (red dots on the right) correlate with elevated PPROM likelihood, whereas lower albumin levels (blue dots on the right) also increase PPROM propensity. This pattern extends to all variables, enabling clinicians to intervene and monitor the impact of the occurrence of PPROM based on indicators from different trimesters. SHAP-based importance analysis captures complex, potentially non-linear interactions and conditional effects learned by the model, which may reveal key characteristics not reflected by simple group-wise statistical tests (Table 2). These features may provide clinical value by supporting risk stratification in specific subpopulations or in combination with other variables. Although they might have not been widely recognized as primary risk factors for PPROM in previous studies, their appearance among the top-ranked characteristics suggests potential relevance to PPROM occurrence or pregnancy outcomes. Identifying such previously underexplored clinical attributes is one of the strengths of AI modeling framework.

**Table 2:** The Accuracy, Precision, Recall, F1-score, and AUC of each model after optimization

Model	Accuracy	Precision	Recall	F1-score	AUC
SVM	0.797	0.800	0.797	0.797	0.864
MLP	0.762	0.744	0.800	0.771	0.844
KNN	0.710	0.770	0.710	0.740	0.814
RF	0.901	0.930	0.868	0.898	0.949
XGBoost	0.922	0.923	0.923	0.923	0.952

## Discussion

Preterm premature rupture of membranes (PPROM) remains a severe pregnancy complication with significant clinical challenges. Current conservative management strategies—encompassing tocolysis, antibiotics, and corticosteroids—demonstrate limited efficacy in significantly prolonging gestation, as approximately 90% of patients deliver within one week of membrane rupture^15^. This reality, coupled with the lack of reliable prenatal risk stratification methods and ongoing controversies in clinical management, underscores the critical need for strategies enabling the timely identification of high-risk cases. Notably, this model identifies imminent risk in late pregnancy rather than providing long‑term prediction of PPROM.

The contemporary understanding of PPROM pathogenesis has evolved beyond a purely mechanical model towards a multifactorial interaction framework, emphasizing dysregulation in collagen metabolism, programmed cell death, and infection-inflammatory cascades. The frequent presence of subclinical chorioamnionitis in 15–25% of PPROM cases highlights the central role of infection-inflammatory pathways, yet reliable biomarkers for detecting intrauterine infections remain elusive^16^.

Our AI-driven investigation has identified critical roles of biomarkers associated with inflammation and oxidative stress in PPROM, including major contributing features such as C-reactive protein (CRP) and albumin, as well as features with modest importance such as uric acid, blood glucose, and ALP (alkaline phosphatase). Notably, maternal serum CRP levels measured at 34–36^+^^6^ weeks emerged as the most influential feature, dynamically reflecting systemic inflammatory burden. Other significant correlators included white blood cell count, lymphocyte percentage, and absolute monocyte count.

The contribution of CRP to PPROM pathogenesis likely occurs through two primary routes. Firstly, via the ascending infection route, microorganisms breach cervical barriers, triggering localized inflammation within the fetal membranes. This inflammatory milieu activates matrix metalloproteinases (MMPs) and generates reactive oxygen species (ROS), ultimately leading to collagen degradation and membrane weakening^17^. Secondly, hematogenous spread allows systemic inflammation to propagate through the placental circulation, inducing complement activation and cytokine storms that accelerate extracellular matrix remodeling and compromise membrane integrity^18,19^.

PPROM is also strongly associated with a state of oxidative imbalance. Studies consistently demonstrate reduced plasma vitamin C levels and elevated total oxidative status (TOS) in pregnancies complicated by PPROM. ROS directly inflict damage upon amniotic cells, further destabilizing membrane integrity^20^. Interestingly, even subdiabetic hyperglycemia may contribute to membrane destabilization through oxidative stress mechanisms, potentially establishing a vicious cycle involving hyperglycemia, heightened oxidative stress, and insulin resistance^21^.

Uric acid appears to potentiate this process by enhancing ROS generation, potentially through activation of the RAS system and NF-κB signaling pathways, thereby exacerbating the inflammatory response^22^. ALP, acting as a potential bridge between inflammation and oxidative stress, may modulate ROS production or influence antioxidant enzyme activity.

Conversely, albumin exerts protective effects through its antioxidant, anti-inflammatory, and nutritional support functions. Its depletion in the context of PPROM may amplify membrane damage susceptibility^23^. Declines in prealbumin and total protein levels likely reflect worsening maternal nutritional status, which could further impair the intrinsic capacity for membrane repair^23^.

The AI model developed in this study achieved a high accuracy of 0.922 utilizing readily available, routine antenatal laboratory parameters, effectively eliminating the need for additional invasive diagnostic testing. This non-invasive approach holds significant potential for enhancing patient compliance and reducing healthcare costs. Our findings strongly underscore the centrality of the inflammation-oxidative stress interplay in PPROM pathophysiology and, importantly, identify novel clinical correlators such as urine specific gravity and thyroid-stimulating hormone (TSH) levels, which warrant further investigation.

Oxidative stress represents not only a key pathological driver but also an actionable target for PPROM management. Clinically, this can be leveraged in several ways: (1) Risk stratification: Incorporating oxidative stress biomarkers (e.g., total oxidant status, antioxidant vitamins) with inflammatory markers could refine risk assessment models for high-risk pregnancy identification. (2) Targeted intervention: While evidence is mixed, antioxidant supplementation (e.g., vitamins C and E) has been explored for PPROM prevention or latency prolongation, offering a potential nutritional strategy^24^. Managing conditions like gestational diabetes to mitigate hyperglycemia-induced oxidative stress is also crucial. (3) Disease monitoring: Dynamic assessment of systemic or local oxidative balance may help in detecting subclinical chorioamnionitis or forecasting labor onset, informing delivery timing.

Translating our multiparameter model into practice requires moving beyond isolated biomarkers to a composite risk score. This integrative approach handles complex scenarios—such as elevated CRP with otherwise normal parameters, or vice versa—by weighing all factors simultaneously. For instance, a high CRP’s contribution is context-dependent and can be offset by strong protective factors like high albumin. Thus, clinical action should be triggered by the overall risk score exceeding a validated threshold, warranting intensified surveillance (e.g., more frequent monitoring and counseling), irrespective of the dominant biomarker driver. To facilitate clinical translation, we deployed our model as a tool with a graphic user interface (Fig. S2) for late-gestation imminent risk stratification. Future work is needed to focus on defining these clinical action pathways and prospectively validating decision thresholds.

Implementing biomarker-based strategies, particularly in resource-limited settings, requires cost-conscious approaches. Although the cost of measuring important features (e.g., CRP and albumin) is low in China, it could be expensive in some low- and middle-income countries (LMICs). Considering the benefits of potential timely interventions and the substantial economic and health burden of PPROM, investment in this risk-stratification approach might still be a rational and economically sound strategy. Further socioeconomic investigation of depth is warrantied for translating our findings into global clinical practice.

This study has several limitations. Its retrospective, single-center design may limit generalizability, necessitating external multi-center validation. Importantly, key sociodemographic and lifestyle confounders were not systematically included, which may restrict the model’s comprehensiveness and causal inference. Future prospective studies should integrate such data, alongside multi-omics approaches and the search for earlier predictive markers, to enable preventative interventions.

Our AI model, based on routine antenatal biomarkers, demonstrated strong performance (AUC = 0.922) for PPROM. This non-invasive, cost-effective approach enables timely, data-driven risk assessment using readily available data, reducing the need for invasive testing and improving patient compliance. The findings confirm inflammation-oxidative stress pathways as key drivers of PPROM, offering mechanistic insights for risk mitigation. Future efforts should focus on multi-center validation and identifying clinically actionable markers detected in earlier gestation to enable early prediction and further enhance risk management strategies, especially in high-risk pregnancies. We further recommend the widespread implementation and shared application of this risk-stratification tool across diverse medical institutions worldwide. Consolidating multi-center clinical data to construct a larger unified database will greatly facilitate the optimization of machine learning algorithms and contribute to the development of more powerful and generalizable risk-assessment models in subsequent research.

## Methods

### Diagnostic criteria for PPROM

PPROM was diagnosed following the ACOG (2013) guideline^25^. The diagnostic criteria are summarized briefly as follows: 1. Essential criteria: persistent or recurrent vaginal fluid leakage or perineal wetness. 2. Definitive diagnosis: meeting the essential criteria and direct visualization of clear amniotic fluid leaking from the cervical os or pooled in the posterior vaginal fornix during a sterile speculum examination. 3. Adjunctive diagnostic tests: indicated when speculum examination is inconclusive. Diagnosis is supported by a positive result on one or more of the following tests performed on vaginal fluid: nitrazine test: vaginal fluid pH > 7.0; fern test: microscopic identification of a characteristic ferning pattern; biomarker test: detection of amniotic fluid-specific proteins (e.g., IGFBP-1). This study was conducted in accordance with the Declaration of Helsinki. Ethical approval for this study was obtained from the Ethics Committee of Shanghai First Maternity and Infant Hospital (Approval Number: KS22331). Prior to analysis, all datasets were rigorously anonymized to safeguard patient privacy. Given its retrospective design and full anonymization of data, the requirement for informed consent was waived by the ethics committee.

### Feature selection

A total of 2667 multidimensional characterization variables of pregnancy were included in this study, covering the continuous obstetric monitoring data of pregnant women after they were filed in Shanghai First Maternity and Infant Hospital. In order to establish a time-series feature analysis framework, a data-driven gestational week segmentation strategy was used: based on the density analysis of gestational week distribution of each type of examination for all pregnant women, combined with the clinical PRACTICE consensus, each type of examination was deconstructed into a number of gestational week window periods. Cross-window period feature importance analysis was performed by machine learning models to accurately identify the key risk factors affecting the occurrence of PPROM at different gestational stages and their dynamic evolution patterns.

During the screening process for each indicator, we performed the following operations to ensure the validity and usability of the final candidate features. We first excluded all the indicators of which gestational weeks were after 36 ^+ 6^ weeks, in order to ensure that all input features included in the model were clinically valuable. We further excluded the indicators for which the missing percentages were higher than 80%. For variables with sparse missing values, we used chained equations (MICE)^26^ for interpolation (Fig. 2), where Bayesian ridge regression methods were used for continuous variables^27^, and logistic regression methods were used for categorical ones^28^. Subsequently, variables with high correlation were screened based on Pearson’s correlation coefficient (Fig. 3a) (normally distributed variables) or Spearman’s correlation coefficient (other variables) (correlation coefficients of >0.9 or <−0.9 were generally specified as correlated). For highly correlated variables, one was kept for further analysis. And finally, the linear model was analyzed using Lasso regression with an L1 Regularization added to the least-squares loss function^29^, which allowed for feature selection to be achieved, yielding final 75 features incorporated into the model (Table 1).

### SHAP interpretability analysis

In order to enhance the interpretability of the model, we further introduced the SHAP (SHapley Additive exPlanations) method for interpretive analysis of the trained XGBoost model. SHAP is one of the most widely used methods for interpreting model outputs of machine learning models, and it is very suitable for the XGBoost used in this study, which is a complex integrated model. It is very applicable and is particularly valuable for clinical risk assessment in the medical field^30^. The application of SHAP analysis in this study is important in several ways. First, it helps us to identify the key clinical variables that play a decisive role in the model, which improves the comprehensibility and credibility of the model results and reduces the barriers to the clinical use of “black-box models”^31^. Second, by combining the distribution of eigenvalues and the directionality of SHAP values, we were not only able to understand which variables were important, but also able to explain the direction of the influence of high or low values of the variables on the model outputs, which is a direct guide for clinical interventions^32^. Third, our work innovatively and systematically applied the SHAP method to the PPROM risk stratification task, which is still relatively rare in existing related studies, and provided a visual and explanatory analytical pathway for clinical identification of high-risk pregnant women.

Therefore, SHAP interpretable analysis enhances the connection between the model and clinical practice, makes the decision-making process of complex machine learning models more transparent, and provides physicians with a basis and confidence in the practical application of the model. This work not only enhances the scientific validity and applicability of the model but also provides a new paradigm and reference for data-driven clinical risk stratification.

### Model development

From the above work, we obtained a clinical dataset of 114,601 data containing 75 features. The construction of the machine learning models was done on PyCharm, which contains many machine learning-related software packages that allow easy implementation of various machine learning functions. In terms of model selection, in order to provide a comprehensive and robust evaluation of the machine learning algorithms selected for the PPROM risk stratification task, we initially selected SVM, MLP, KNN, RF, and XGBoost as the candidate base models, which represent different algorithmic ideas and have a good basis for application in medical risk assessment research. SVM is a geometric algorithm that finds the optimal SVM is a geometric algorithm that can find the optimal hyperplane for data segmentation^33^. MLP is a feed-forward neural network, which belongs to the type of neural network with strong nonlinear modeling ability^34^. KNN belongs to the distance model, where the class of the new samples is determined by the k closest training sample points according to the classifying decision rule^35^. RF^36^ and XGBoost belong to the tree model, while XGBoost integrates the Boosting method, which is more general and flexible^37^.

As the proportion of positive and negative samples in the experiment varied considerably, and out of the 114,601 data, only 1895 had PPROM, we first divided the non-PPROM data into ten portions, added the PPROM data to each portion, and then removed the non-PPROM data to a proportionate amount of the PPROM data by using Random Under-sampling^38^. Such a strategy formed ten reasonable datasets of 3850 data each, and each sample was randomly divided into 90% of the training set and 10% of the test set during training. Receiver operating characteristic (ROC) is an important tool used for binary classification model evaluation, the area under the ROC is the AUC, the larger the AUC, the higher the model accuracy. Accuracy is the proportion of normally classified samples, precision is the proportion of truly positive samples among the predicted positive samples, recall is the ability of the model to recognize positive samples, and the F1 score is the weighted summed mean of precision and recall. In selecting the base model, the model is evaluated by five metrics together, namely, AUC, Accuracy, Recall, Precision, and F1 score, with AUC as the main evaluation metric.

### Statistical analysis

Statistical significance was defined as a two-sided *P* value < 0.05. Continuous variables are presented as mean ± standard deviation, while categorical variables are expressed as frequency and percentage. The normality of continuous variables was assessed using the Kolmogorov–Smirnov test. For group comparisons, the independent samples t-test was applied to normally distributed continuous variables, whereas the Mann–Whitney U test was used for non-normally distributed continuous variables. Categorical variables were compared using the chi-square test. Correlation analyses were performed using Pearson’s correlation coefficient for normally distributed continuous variables or Spearman’s rank correlation coefficient for non-normally distributed or ordinal variables. Variables with an absolute correlation coefficient greater than 0.90 were considered to be highly correlated.

## Supplementary information

Supplementary Information.

## Data Availability

The data used in this study are derived from the antenatal registration system of Shanghai First Maternity and Infant Hospital. Due to institutional and ethical restrictions regarding patient privacy, the data are not publicly available. Researchers seeking access to the data may contact the corresponding author; access will require approval from the institutional data governance committee. Please contact Jinjin Li (lijinjin@sjtu.edu.cn) with inquiries regarding data. A synthetic dataset maintaining the statistical properties of the original data is available at https://github.com/handadangjia/PPROMPrediction.git.

## Code availability

Data processing, modeling, and analysis were conducted under Python version 3.8. The main libraries used include the modeling tool XGBoost (version 2.1.4), scikit-learn (version 1.3.0), the interpretability analysis tool SHAP (version 0.44.1), and the plotting tool matplotlib (version 3.5.1). The code was developed and run in the PyCharm IDE on the Windows 10 operating system. The PPROM risk stratification tool is implemented in Python and provides a GUI for clinical feature input, XGBoost classification, and SHAP explainability. The code is openly available at https://github.com/handadangjia/PPROMPrediction.git.
