# Supplementary material for: Noninvasive PPROM risk stratification with explainable AI using routine antenatal CRP and albumin
Source: NPJ Digit Med. 2026 Jun 10;9:718. doi: 10.1038/s41746-026-02884-z (PMC13598096; doi:10.1038/s41746-026-02884-z)
Supplement: Supplementary file 1 [file 41746_2026_2884_MOESM1_ESM.pdf]

SUPPLEMENTARY FIGURES

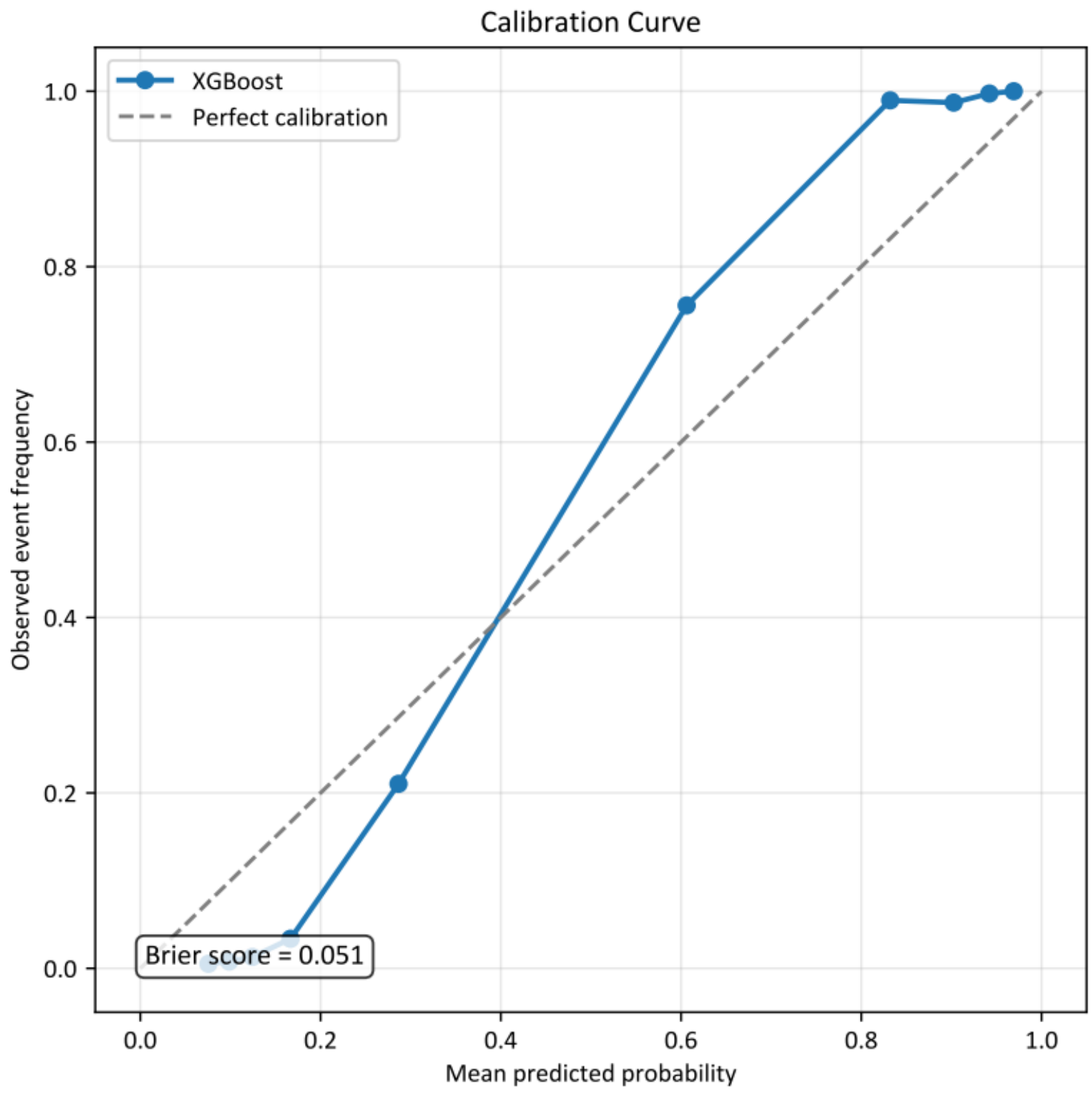

Figure S1. Calibration analysis for the trained model.

The close alignment between the calibration curve and the ideal line indicates well-calibrated probability risk estimation.

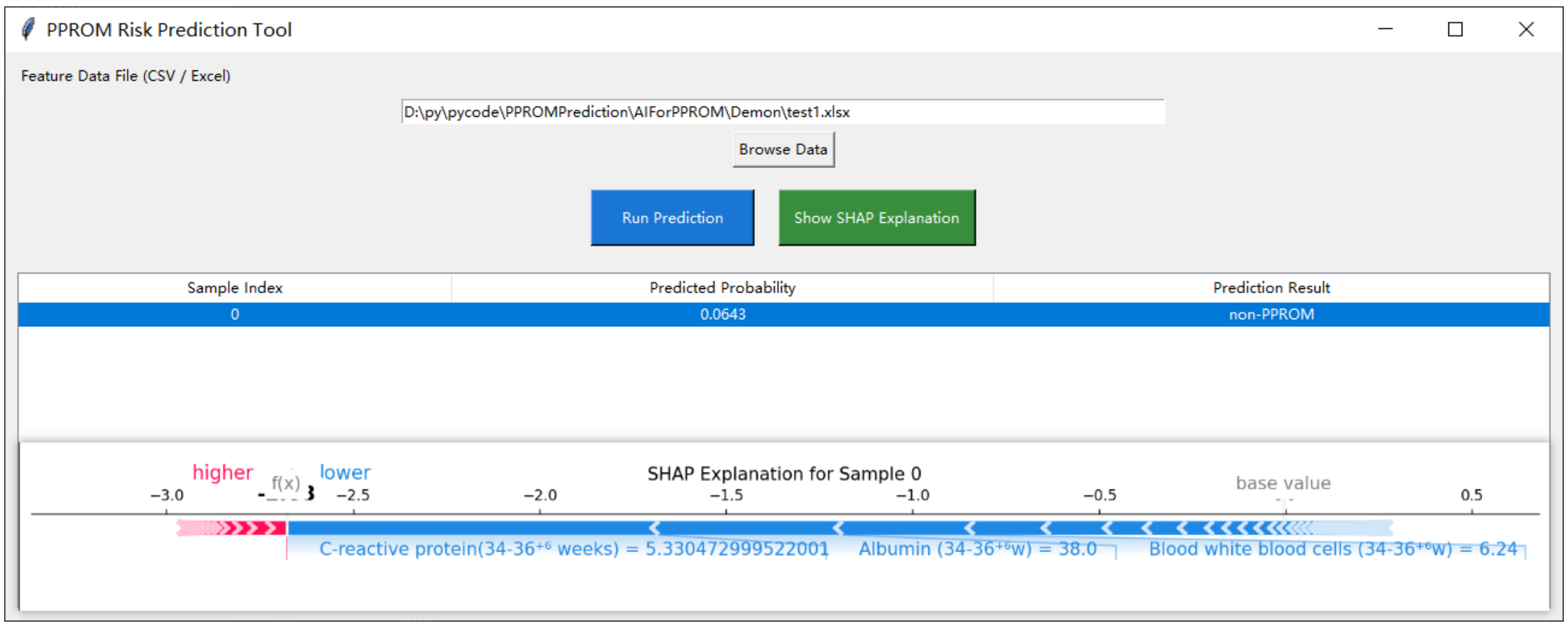

Figure S2. GUI of PPROM risk stratification tool.

Interface of the risk assessment tool allowing clinicians to upload patient data and receive PPROM risk estimates along with SHAP explainability.
